# Myocardial Ischemia/Reperfusion Injury: Molecular Insights, Forensic Perspectives, and Therapeutic Horizons

**DOI:** 10.3390/cells14191509

**Published:** 2025-09-27

**Authors:** Maria Sofia Fede, Gloria Daziani, Francesco Tavoletta, Angelo Montana, Paolo Compagnucci, Gaia Goteri, Margherita Neri, Francesco Paolo Busardò

**Affiliations:** 1 Department of Biomedical Sciences and Public Health, Section of Legal Medicine, University Politecnica delle Marche, 60126 Ancona, Italy; maria.sofia.fede@pm.univpm.it (M.S.F.); g.daziani@pm.univpm.it (G.D.); francescotavoletta@gmail.com (F.T.); a.montana@univpm.it (A.M.); 2 Cardiology and Arrhythmology Clinic, Marche University Hospital, 60126 Ancona, Italy; paolocompagnucci1@gmail.com; 3 Institute of Pathologic Anatomy and Histopathology, University Politecnica delle Marche, 60126 Ancona, Italy; g.goteri@staff.univpm.it; 4 Section of Forensic Pathology, Morphology, Surgery and Experimental Medicine Department, University of Ferrara, 44121 Ferrara, Italy; nremgh@unife.it

**Keywords:** myocardial ischemia reperfusion injury, cardiac oxidative stress, cardiac inflammation, myocardial revascularization, percutaneous cardiac intervention, cytokines, forensic cardiopathology, cardioprotective therapies

## Abstract

Acute myocardial infarction (AMI) remains the leading cause of death worldwide, with myocardial ischemia/reperfusion injury (MIRI) emerging as a significant factor influencing patient outcomes despite timely reperfusion therapy. MIRI refers to paradoxical myocardial damage that occurs upon restoration of coronary blood flow and is driven by complex inflammatory, oxidative, and metabolic mechanisms, which can exacerbate infarct size (IS), contributing to adverse outcomes. This review explores the molecular and cellular pathophysiology of MIRI, emphasizing both its clinical and forensic relevance. The principal mechanisms discussed include oxidative stress and mitochondrial dysfunction, calcium overload and ion homeostasis imbalance, inflammatory responses, with particular focus on the NLRP3 inflammasome and cytokine pathways, and multiple forms of cell death (apoptosis, necroptosis, pyroptosis, and autophagy). Additionally, the authors present original immunohistochemical findings from autopsy cases of patients who suffered ST-segment elevation myocardial infarction (STEMI) and underwent percutaneous coronary intervention (PCI), but subsequently died. These findings underscore that successful reperfusion does not completely prevent delayed complications, like arrhythmias, ventricular fibrillation (VF), and sudden cardiac death (SCD), often caused by secondary MIRI-related mechanisms. Moreover, the case series highlight the diagnostic value of inflammatory markers for pathologists in identifying MIRI as a contributing factor in such fatalities. Finally, immunotherapeutic strategies—including IL-1 and IL-6 inhibitors such as Canakinumab and Tocilizumab—are reviewed for their potential to reduce cardiovascular events and mitigate the effects of MIRI. The review advocates for continued multidisciplinary research aimed at improving our understanding of MIRI, developing effective treatments, and informing forensic investigations of reperfusion-related deaths.

## 1. Introduction

AMI is a major global health issue and still represents the single most prevalent cause of death worldwide, despite continuous advances in AMI prevention and treatment [1]. Myocardial infarction (MI) is also a widespread occurrence in forensic medicine, with concerns related to both the differential diagnosis with other cardiovascular conditions and the correct dating of the disease [2,3]. AMI is defined as cardiomyocyte necrosis in a clinical setting consistent with acute myocardial ischemia, as identifiable through a series of clinical (prolonged chest pain), biochemical (elevated cardiac troponin levels), and instrumental (ECG and/or regional wall motion abnormalities) parameters [4]. Ischemia is mainly caused by acute coronary atherothrombotic occlusion or mural thrombus (MI type 1), but it could also be due to other mechanisms reducing coronary flow and/or oxygen supply (MI type 2–5), as recently updated in the fourth universal definition of myocardial infarction, published in 2018 by the Joint Task Force of the European Society of Cardiology, the American College of Cardiology Foundation, the American Heart Association, and the World Heart Federation [5]. This condition of tissue hypoxia triggers specific metabolic (arrest of aerobic metabolism and onset of anaerobic glycolysis with progressive accumulation of catabolites), inflammatory, and oxidant changes, leading to evolving cardiac cell damage—from reversible injury to irreversible death—followed by cardiac repair [6].

The early restoration of the coronary blood flow (i.e., reperfusion therapy) brought by thrombolytic therapy or primary percutaneous coronary intervention (PPCI) is key to save myocardial tissue from ischemic death in patients with STEMI [7], because coronary artery recanalization provides both the oxygen restoration and the availability of inflammatory cells, which can initiate cardiac tissue healing. Nonetheless, it has been known for more than 60 years that reperfusion itself can paradoxically contribute to cardiomyocyte death (through apoptosis, necrosis, necroptosis, and pyroptosis), energy metabolism disorders (mainly due to mitochondrial alterations, linked to oxidative stress and calcium overload), additional inflammatory response, and endothelial cells’ death with coronary microvascular dysfunction. These events occur within the first few minutes of reperfusion, leading to an increase in the final IS, which involves residual viable myocytes and causes additional damage, referred to as MIRI [8]. A comprehensive understanding of the pathogenesis and timing of MIRI could lead to the development of new, effective therapeutic strategies to limit the progression to heart failure [9]. In a very recent expert consensus document, published last year from the Canadian Cardiovascular Society, a new classification of atherothrombotic MI with reperfusion therapy is proposed, specifically based on progressive tissue changes in the myocardium due to ischemia/reperfusion injury (IRI), aiming for the development of new tissue-injury-specific therapies for MI [10]. Starting with the CANTOS Trial in 2017, which was the first clinical study able to demonstrate the successful secondary prevention of cardiovascular events by targeting the immune system, especially pro-inflammatory cytokines [11], scientific research intensified the study of the pathobiology of MIRI. Research efforts focused on the role of cardiac oxidative stress and immuno-inflammatory mechanisms, aiming to identify more and more suitable pathways to be targeted by novel therapies, either physical or pharmacological ones, with the final aim being to combine benefits derived from myocardial reperfusion with those from these cardioprotective strategies, with a potentially synergistic action [12]. More importantly, for the forensic pathologist, understanding MIRI may also be key in recognizing that unfavorable outcomes and even death may occur despite successful and early reperfusion, with clear implications for malpractice claims. Finally, the cellular and plasma factors involved in MIRI might be used as immunohistochemical markers of ischemic myocardial damage, therefore representing an additional diagnostic tool for pathologists, allowing them to confirm MIRI as a potential cause of death.

In this article, pathophysiological mechanisms involved in MIRI after AMI are reviewed at the molecular and cellular level, aiming to provide a valid reference for both clinicians and forensic pathologists. In addition, the authors will firstly present a collection of myocardial explicative histological images collected from autopsy cases of AMI death after successful reperfusion, but also potentially MIRI cases after cardiac arrest (and other ischemia situations, without acute infarction), analyzing cardiac expression of inflammatory markers and morphological changes, and secondly a brief focus on the results of recent clinical trials with immunotherapeutic strategies targeting MIRI. The ultimate goal is to provide an up-to-date state-of-the-art overview of the knowledge on the mechanisms underlying MIRI, potential therapeutic interventions, and the forensic implications of this emerging and complex phenomenon.

## 2. MIRI and Oxidative Stress: Mitochondria and Calcium Overload

When oxygen supply is restored with reperfusion after a period of myocardial ischemia, there is an increased production of free radicals from oxygen (reactive oxygen species, ROS) which may also react with nitrogen, producing reactive nitrogen species (RNS). ROS and RNS exhaust ROS scavengers and other antioxidants molecules, resulting in the progressive accumulation of oxidants, which may lead to cell damage and release of inflammatory factors [13,14,15].

Oxygen reduction yields superoxide (O_2_^−^) anion, which can determine the hydroxyl (OH) radical formation through two different pathways: one involving spontaneous combination with hydrogen peroxide (H_2_O_2_), while the other consisting on the Fenton reaction. The RNS group includes nitric oxide (NO), nitrogen dioxide (NO_2_), and peroxynitrite (ONOO^-^) [14]. During MIRI, sources of ROS include NADPH oxidase, xanthine oxidase, nitric oxide synthase (NOS), and mitochondria [14,16,17]. NADPH oxidase is an enzyme that generates superoxide and consists of a membrane-bound catalytic subunit (NOX) along with several cytosolic regulatory subunits. NOX2 serves as the catalytic subunit of phagocyte NADPH oxidase. Upon activation, the cytosolic components migrate to the transmembrane catalytic protein gp91phox, forming a functional NADPH oxidase complex. Researchers have shown that NADPH oxidase is expressed in cardiomyocytes. NADPH oxidase is a critical source of ROS, which subsequently influences the redox state of the myocardium. However, the most relevant process for ROS generation consists in the production of O_2_^−^ anion mediated by the mitochondrial succinate, which activates the REarranged during Transfection (RET) at the level of Complex I [18,19]. O_2_^−^ anion is released into the matrix by Complex I, and in the intermembrane space by Complex III [18].

Several cellular and molecular pathways are activated by ROS, leading to cell death, both through apoptosis and pyroptosis. Shen et al. demonstrated the direct linkage between these free radicals and the activation of the nucleotide-binding oligomerization (NOD)-like receptor protein 3 (NLRP3) inflammasome pathway (enhancing both inflammatory response and pyroptosis), after ROS generation by uric acid [20].

Moreover, ROS are the main species responsible for mitochondria dysfunction, and they could also lead to intracellular calcium overload. Indeed, during MIRI, ROS are an important trigger of the opening of the mitochondrial permeability transition pore (mPTP) [16,21], which promotes the escape of Cytocrome C (CytC) in cytosol [22]. As demonstrated by Schriewer et al. [23], mPTP’s opening requires ROS presence and can also in turn support ROS production; they showed that another molecule involved in the process of mitochondria-mediated necrosis during ischemia/reperfusion (I/R) was poly (ADP-ribose) polymerase-1 (PARP). PARP can participate in oxidative stress amplification and mediate cell death due to oxidative damage after I/R. During I/R, PARP could participate in mPTP opening through poly ADP-ribosylation of the mitochondrial target, and in turn mPTP opening could enhance PARP activity. The consequentiality of the events is not yet well understood, and these authors proposed PARP inhibition as a possible actionable target to alleviate reperfusion injury even when mPTPs are already opened [23].

**Figure 1:**
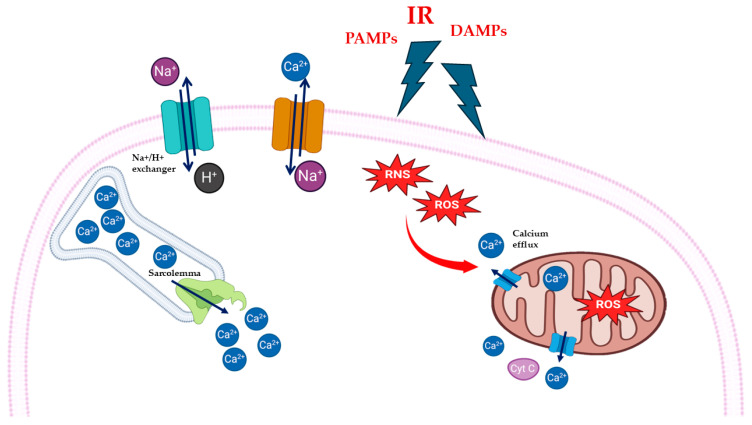
Schematic representation of cardiac oxidative stress and calcium overload during myocardial ischemia/reperfusion injury (MIRI). Reperfusion injury (IR), pathogen-associated molecular patterns (PAMPs), and damage-associated molecular patterns (DAMPs) cause increase in intracellular reactive oxygen species (ROS) and reactive nitrogen species (RNS). Oxidative stress causes release of calcium ion (Ca^2+^) form mitochondria and sarcolemma through Sarco-Endoplasmic Reticulum Calcium ATPase (SERCA Ca^2+^-ATPase).

One of the effects of the opening of mPTP channels is the release of Ca^2+^ ions. Calcium overload is a typical event that occurs because of reperfusion and contributes to cardiac cells damage; the increased cytosolic concentration of Ca^2+^ ions may lead to apoptosis [24]. Initially during the ischemic phase, the myocardial cells decrease the aerobic glycolysis and Krebs cycle, resulting in a switch to anaerobic metabolism, especially anaerobic glycolysis, which lead to the accumulation of lactate and H^+^ ions, causing a decrease in intracellular pH [25,26]. In this setting of intracellular acidosis, the activation of the Na^+^/H^+^ exchanger (NHE), which extrudes one H^+^ in exchange for one Na^+^ entering the myocyte, results in the cytosolic accumulation of Na^+^. Furthermore, during ischemia, the production of ATP is curtailed, determining the inhibition of both Na^+^/K^+^-ATPase [27], and Sarco-Endoplasmic Reticulum Calcium ATPase (SERCA Ca^2+^-ATPase) [28], which in turn leads to an increase in intracellular Na^+^ (Figure 1).

During reperfusion, the restoration of oxygen levels promotes normalization of extracellular pH and reactivation of mitochondrial respiration, so the NHE continues to support the H^+^ efflux from the cells with a persistent increase in intracellular Na^+^, which in turn activates the Na^+^/Ca^2+^ exchanger (NCX) reverse mode, favoring intracellular Ca^2+^ accumulation [16,28,29,30,31]. Another mechanism that causes an increase in circulating free Ca^2+^ is related to the sarcoplasmic reticulum (SR). The efficient transfer of ATP to sarcoplasmic reticulum (SR) causes Ca^2+^ oscillations, due to accumulation and release of Ca^2+^ by SR. The released Ca^2+^ can be intercepted by adjacent SRs, causing the propagation of calcium waves, and hypercontracture, which can lead to cell death [15,23,28,32].

The increased production of ROS, due to the renewed oxygen supply to tissues, is thus one of the main factors that interferes with proper cellular homeostasis. These highly reactive molecules interact at different levels and with different pathways, causing cellular dysfunction. A clear example of this is the mitochondria, whose damage is, in turn, capable of sustaining ROS production.

**Figure 2:**
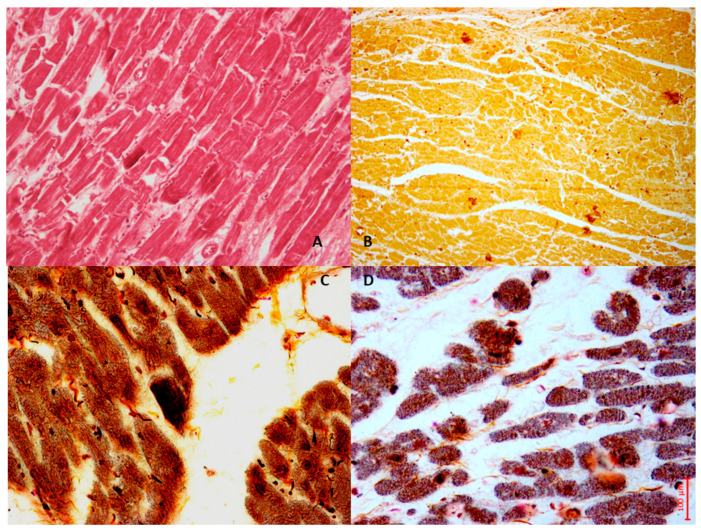
The case of a 61-year-old man who died six days after experiencing an acute heart ischemia, which was treated with PCI for acute STEMI. In the bright-field microscopy image (**A**), we observe the left ventricles stained with Hematoxylin and Eosin. Image (**B**) illustrates NOX2 expression in the cardiac tissue, demonstrating moderate immunopositivity to the anti-NOX2 antibody (Santa Cruz, CA, USA). Additionally, the Von Kossa stain, a histochemical technique that highlights calcium, reveals prominent calcium deposits in the anterior myocytes, showing needle-like crystal formations in the bright-field images ((**C**) phase contrast field and (**D**) bright light field).

In the following sections, histomorphological images are showed illustrating the phenotypic outcomes resulting from pathway alterations related to oxidative stress and calcium overload in MIRI. In particular, Figure 2 depicts a case based on personal observation of a 61-year-old man with chronic hypertension who presented in an emergency room with squeezing retrosternal chest pain and dyspnea. Elevated cardiac enzymes and electrocardiography results suggested acute STEMI since the hospital performed a quick PCI. A control ECG showed nearly complete resolution of the ST-segment elevation. Six days after the treatment, the patient suffered a sudden cardiac arrest due to VF and died.

## 3. MIRI, NLRP3 Inflammasome, and Cytokines: From Homeostasis to Dysregulation

Inflammasomes are multiprotein complexes involved in the inflammatory response [33], interacting with damage- or pathogen-associated molecular patterns (respectively, DAMPs or PAMPs) [34]. Inflammasomes are a large and heterogenous group of molecules. Regarding their role in MI, the best characterized inflammasome is the nucleotide-binding oligomerization domain (NOD), leuchine-rich repeat (LRR), and pyrin domain (PYD)-containing protein 3 (NLRP3), which also plays a role in the inflammation cascade during MIRI [33,34]. The NLRP3 inflammasome is composed of the NLRP3 protein, apoptosis-associated speck-like protein (ASC), and pro-caspase-1. The NLRP3 protein can be in turn divided into three domains: NATCH, a central nucleotide domain mediating oligomerization, a C-terminal LRR domain, and a PYD [34]. A PYD is also present in ASC, combined with the caspase recruitment domain (CARD) [34,35,36].

The expression of the NLRP3 inflammasome can be regulated through Nuclear Factor (NF)-kβ, which is activated by Toll-like receptor 4 (TLR4) once it recognizes tissue damage or a signal of infection (DAMPs or PAMPs). After its activation, NF- kβ in turn also promotes the expression of pro-IL-1β and pro-IL-18, which are then activated by caspase-1, a product of NLRP3 action on pro-caspase-1. IL-1β and IL-18 enhance the inflammatory response by increasing cytokines’ production, immune response, and extracellular matrix’s turnover [37]. Consistently, in an in vitro model of MIRI, Huang et al. demonstrated the overexpression of the NLRP3 inflammasome by cardiac fibroblasts, with a consequent increase in cellular levels of IL-1β, IL-18, TNF-α, NLRP3, caspase-1, and ASC after hypoxia/reoxygenation (H/R) [38].

Knowledge about NLRP3 has recently widened, with the identification of novel and increasingly complex homeostatic mechanisms interacting with the inflammasome.

Given this increasingly recognized complexity, it comes as no surprise that preclinical studies on the modulation of the inflammasome pathway yielded contrasting results on the attenuation of MIRI. In 2011, Kawaguchi et al. observed that in a mouse model of MIRI, knockout (KO) mice for ASC and caspase-1 showed a less extensive infarcted area and a reduction in cytokines and interleukins’ expression compared with the wild-type (WT) mice. Furthermore, the authors provided evidence that ASC and caspase-1 were expressed in the myocardial infarct site [39]. Another study confirmed that the NLRP3 inflammasome is upregulated in the myocardium after MI, and that its deficiency improves myocardial function. Sandanger et al. demonstrated that, in rats and mice models of MIRI, myocardial fibroblasts express TLRs 1–4 and 9 [40]. In support of the involvement of NLRP3 in the inflammatory response following I/R, their study showed that KO mice for NLRP3 maintain contractile function and coronary flow in the heart and have a smaller infarct zone than WT mice, in an ex vivo experiment [40]. However, the same group later surprisingly observed that KO mice for NLRP3 and ASC had a larger infarct size after I/R injury, leading the authors to hypothesize that the NLRP3 inflammasome might contribute to cardioprotection against MIRI, by interacting with the protective reperfusion injury salvage kinase (RISK) pathway [41], which is a combination of the pro-survival kinase signaling cascades phosphatidylinositol-3-OH kinase (PI3K)-Akt and extracellular regulated kinase (ERK)-1/2 [42].

**Figure 3:**
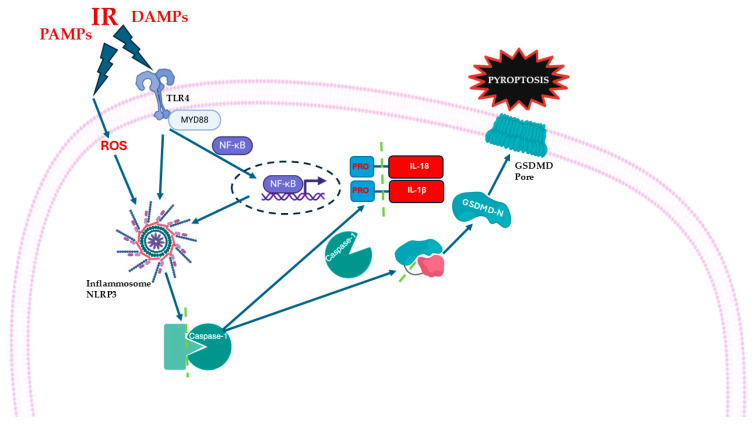
Schematic representation of inflammatory pathways during myocardial ischemia/reperfusion injury (MIRI). Damage-associated molecular patterns (DAMPs) and pathogen-associated molecular patterns (PAMPs) activate Toll-like receptor 4 (TLR4), which recruits the adaptor protein Myeloid differentiation primary response 88 (MyD88), and activates Nuclear Factor kappa B (NF-κB). NF-κB promotes the transcription of pro-inflammatory cytokines, including pro-IL-1β and pro-IL-18. Cellular stress and increased reactive oxygen species (ROS) contribute to the activation of the NOD-like receptor family pyrin domain containing 3 (NLRP3) inflammasome. NLRP3 cleaves pro-caspase-1 into active caspase-1. Caspase-1 then processes pro-IL-1β and pro-IL-18 into the active forms. Caspase-1 also cleaves Gasdermin D (GSDMD), releasing its N-terminal fragment (GSDMD-N), which forms pores in the plasma membrane, leading to cell swelling, membrane rupture, and pyroptosis.

The activation of TLRs by DAMPs and/or PAMPs after myocardial ischemia could enhance the inflammatory response, thus initiating and contributing to MIRI, by activating several molecular pathways other than NLRP3-mediated pathways. Chong et al. detailed the role of TLR4 in MIRI, describing the transmembrane recruitment of the Myeloid differentiation protein (MyD88), and all the distal intracellular signaling leading to inflammation and apoptosis [43]. Many other studies confirmed the central implication of TLR4 signaling in MIRI, focusing on the activation of NF-kβ, and the release of inflammatory factors such as TNF-a, IL-6, and IL-1 [43,44,45,46] (Figure 3).

The involvement of MyD88 in MIRI was demonstrated by several studies in which its inhibition was associated with a decrease in reperfusion damage. In 2020, Miao et al. reported that in a murine model of MIRI, inhibition of MyD88 with TJ-M2010-5 (an experimental drug) resulted in reduced inflammatory response and infarct area [47]; Yang and colleagues also demonstrated that MyD88 inhibition can reduce severe MIRI after heart transplantation in in vivo mice models [48]. MyD88 may interact with other TLRs, in particular TLR9, whose expression is increased during MI [49]. Finally, mitochondrial DNA (mtDNA) may be released in case of significant oxidative stress and activate a similar pathway, resulting in NF-kβ activation [50].

Therefore, there is an astonishing complexity of the inflammatory response during MIRI, due to the multiple cellular and molecular pathways implicated, which may have pleiotropic effects. The NLRP3 inflammasome is seemingly the major activator of the inflammatory response in MIRI, but it might also have a cardioprotective function, complicating the development of therapies targeting this pathway. Further studies will be needed to decipher the homeostatic versus maladaptive role of individual molecules, and to identify novel molecular targets.

**Figure 4:**
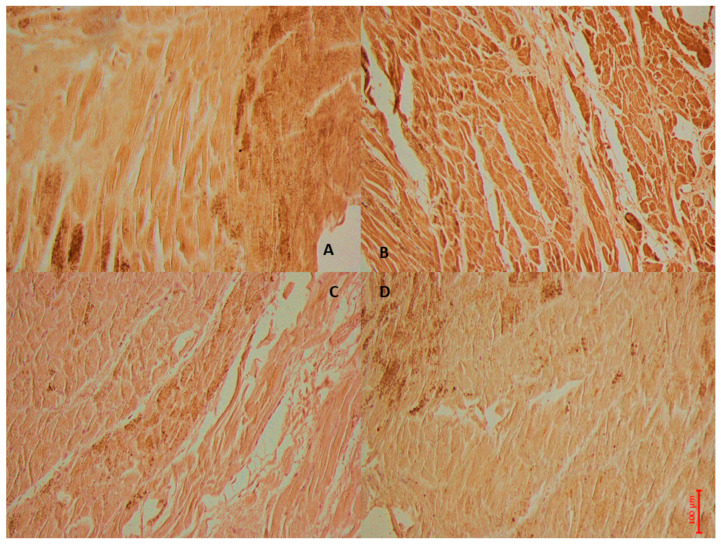
The case of a 59-year-old man who died 2 days after experiencing an acute heart ischemia, which was treated with PCI for acute STEMI. In image (**A**), NF-kβ shows a wide immunohistochemical positive reaction of the left ventricles, while image (**B**) shows strong IL6 expression in the cardiac tissue, and image (**C**) demonstrates moderate immunopositivity to the anti-IL1 antibody. Image (**D**) illustrates a mild reaction to TNF immunoreaction.

In Figure 4, personal observation of a 59-year-old man who presented to the emergency department with acute chest pain is shown. After assessment, he was diagnosed with STEMI. Given the urgency of his condition, he underwent immediate angiography followed by PCI. Initially, after the procedure, he appeared stable, with positive signs of recovery. However, on the second day following PCI, he suddenly became unresponsive and died unexpectedly. An ECG conducted at that time revealed a VF rhythm, indicating a critical arrhythmia associated with his underlying cardiac condition. This severe outcome highlights the complexities and potential complications associated with acute myocardial infarctions and the repercussions of reperfusion therapy.

## 4. MIRI and Apoptosis

The term “apoptosis” indicates a highly organized, evolutionary conserved, genetically regulated pathway for maintaining homeostasis in multicellular organisms and was coined by Kerr, Wyllie, and Currie in 1972 [51]. This process refers to a controlled form of cellular death, wherein the cell undergoes distinct structural modifications like contraction and condensation of cytoplasm and nucleus, with the formation of apoptotic bodies [51]. Caspases, a family of cysteine proteases, are responsible for the breakdown and fragmentation of cytoskeletal and nuclear proteins during the progression of apoptosis [52]. Subsequently, macrophages phagocytize apoptotic bodies, which are recognized due to the presence of externalized phosphatidylserine on the outer leaflet of the bilayer envelope, without triggering any inflammation [53]. Previous studies have highlighted that apoptotic cell death is one of the primary forms of cardiomyocyte death during myocardial infarction and MIRI [54,55]. As widely addressed, MIRI induces an elevation in ROS levels, cellular damage, and cardiovascular dysfunction, ultimately resulting in cardiomyocyte apoptosis [56,57]. The induction of apoptosis occurs via three major signaling pathways: the extrinsic pathway, the intrinsic pathway, and the endoplasmic reticulum stress (ERS) pathway [58]. The extrinsic pathway is triggered by the presence of transmembrane death receptors, which are classified as members of the tumor necrosis factor receptor (TNFR) family and possess the “death domain”. The transmission of death signals from the cell surface to intracellular pathways is facilitated by the coupling of specific ligands and their corresponding death receptors. Among the various ligand–receptor pairings, the most relevant include the apoptosis-stimulating fragment ligand (FasL) and FasR, TNFα, and TNFR1, as well as TNF-related apoptosis-inducing ligand (TRAIL) and either DR4 or DR5 [58]. Binding of ligands with death receptors results in the recruitment of Fas-associated death domain (FADD) and its subsequent binding to the ligand–receptor complex [59]. FADD activates the pro-caspase-8, leading to the formation of a death-inducing signaling complex (DISC) [60]. This complex then activates caspase-8, which subsequently activates caspase-3 and -7. This process triggers the apoptotic cascade response, ultimately resulting in the execution phase of apoptosis [61,62]. It has been shown that caspase-3 overexpression leads to a higher susceptibility to enhanced myocardial injury following coronary artery ligation, wherein the ischemic tissue is reperfused for a duration of 24 h, resulting in an increase in mortality following MIRI [63]. Additionally, the Fas pathway plays a crucial role in mediating cardiomyocyte apoptosis during MIRI [64], and the onset of reperfusion in mouse models of MIRI is accompanied by an increase in TNF-α and TRAIL levels [65].

The intrinsic pathway is activated by hypoxia, hyperthermia, and low levels of growth factors and is triggered by mitochondria. These stimuli trigger the opening of mPTP and markedly reduce the mitochondrial transmembrane potential, ultimately leading to an increased release in pro-apoptotic proteins, including CytC from the mitochondrial intermembrane space to the cytoplasm [66]. Caspase-9 is activated by CytC, resulting in the production of caspase-3 and -7. The B-cell lymphoma 2 (Bcl-2) family proteins situated in the outer mitochondrial membrane are responsible for regulating the mitochondrial-initiated events mentioned above [67]. By controlling the permeability of the mitochondrial membrane, Bcl-2 family’s proteins play a crucial role in regulating the release of CytC. They can be functionally classified as pro-apoptotic and anti-apoptotic proteins. Within the pro-apoptotic group, we find Bad, Bax, Bak, Bid, Bim, Puma, and BNIP3 proteins are included [68]. Interestingly, the extrinsic and intrinsic apoptosis pathways seem to interact: in fact, Fas-mediated apoptosis can lead to mitochondrial damage through the cleavage of Bid by caspase-8 [69]. Overexpression of Bax is observed in the intrinsic apoptosis pathway of ischemic myocardial tissue, and preventing Bax activation has been found to lower apoptosis and enhance MIRI [70]. It was demonstrated that the RISK pathway exerts anti-apoptotic activity during I/R processes by phosphorylating, and therefore inactivating, numerous pro-apoptotic proteins, such as BAD, Bax, BIM, and p53 [71,72,73]. In particular, RISK action on Bax can interfere with its translocation to the mitochondria, thus preventing apoptosis. Remarkably, compelling evidence suggests a correlation between the RISK pathway and the mPTP in rat myocytes. These findings provide evidence that the cardioprotection effect of the PI3K-Akt kinase cascade is achieved through the inhibition of mPTP opening [74].

**Figure 5:**
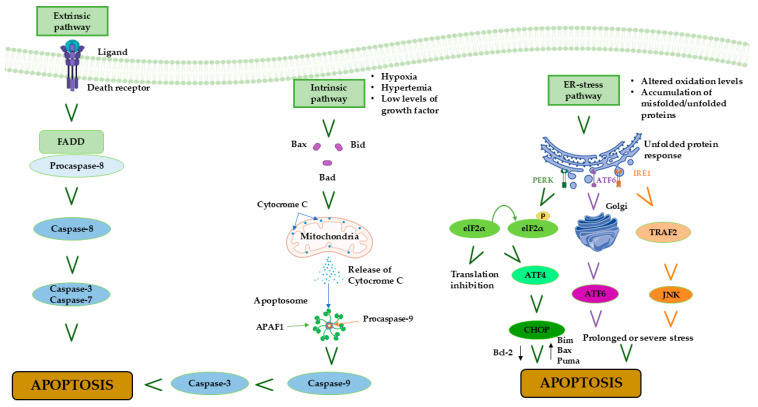
Schematic representation of apoptosis pathways showing the roles of hypoxia, oxidative stress, ER (endoplasmic reticulum) stress, growth factor depletion, unfolded protein response (IRE1, PERK, ATF6), Bim, and pro-caspase-9 in cardiomyocyte apoptosis during myocardial ischemia/reperfusion injury (MIRI).

Finally, the emerging ERS pathway is implicated in a wide range of physiological functions and pathological injuries, encompassing protein folding, intracellular Ca^2+^ storage, oxidative stress, hypoxia, ischemia, and lipid metabolism disorder [75]. ERS plays a crucial role in maintaining cell viability; in fact, not all ERS is harmful. Nevertheless, if it persists for an extended period, it can trigger apoptosis [75]; furthermore, it has been demonstrated that MIRI may be associated with ERS [76]. The presence of unfolded proteins in ERS leads to myocardial injury, which triggers ERS and disrupts the metabolic state of cardiomyocytes, causing greater harm [77]. ERS is amplified during MIRI, and inhibiting ERS has been verified to reduce MIRI [78]. Altered ER oxidation levels result in the abnormal formation of disulfide bonds and the accumulation of peptides. These changes activate intracellular reactions labeled as “the unfolded protein response” (UPR) [79], which leads to the activation of three transmembrane stress sensors: inositol-requiring enzyme 1 (IRE1); activating transcription factor-6 (ATF6) and protein kinase RNA (PKR)-like ER kinase (PERK) [76]. The ERS transcription factor ATF6 initiates a defensive response against MIRI in cardiomyocytes [80,81] (Figure 5).

## 5. MIRI and Necroptosis

**Figure 6:**
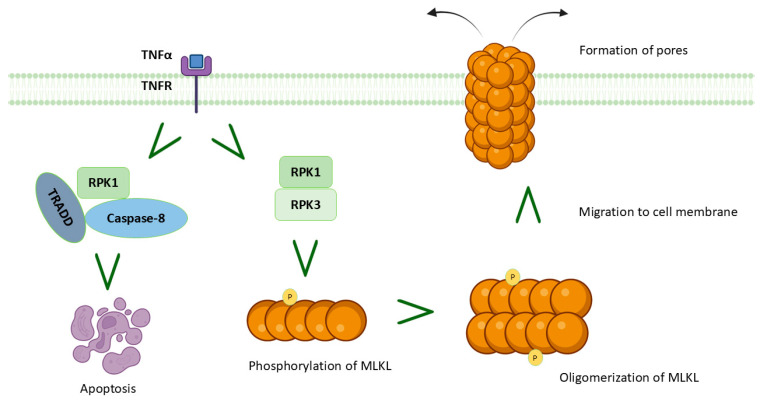
Schematic representation of necroptosis during myocardial ischemia/reperfusion injury (MIRI). Necroptotic pathway in myocardial infarction, illustrating mixed-lineage kinase domain like protein (MLKL) phosphorylation, oligomerization, translocation to the plasma membrane, pore formation, and progression to apoptosis.

Necroptosis represents a unique form of cell regulatory necrosis, displaying both necrotic and apoptotic traits [82]. It is widely known that necrosis, in contrast with apoptosis, occurs without proper regulation and is triggered by external factors like hypoxia or inflammation [83]. Necroptosis often involves the upregulation of various pro-inflammatory proteins and compounds, which leads to the rupture of the cell membrane and the subsequent release of cellular contents into the surrounding areas. Therefore, a chain reaction of inflammation and tissue damage occurs. Necroptosis is implicated in a wide range of physiological functions and disease processes, encompassing protein folding, intracellular Ca^2+^ storage, oxidative stress, hypoxia, ischemia, and lipid metabolism disorder [84]. Morphologically, necroptosis primarily presents itself as cellular enlargement and disruption of the cell’s plasma membrane. In contrast to apoptosis, necroptosis does not depend on caspase activation and instead relies on distinct signaling pathways. This process is characterized by the release of DAMPs and cytokines, thereby triggering an immune-inflammatory response that exacerbates tissue injury [84]. Necroptosis can arise from various triggers, with TNF-α induced necroptosis being the most prominent. This form of necroptosis is primarily facilitated by receptor interacting protein kinases (RIPK)1 and 3. The interaction between RIPK1 and RIPK3 occurs via the shared RIP homotypic interaction motif (RHIM), resulting in the formation of necrosomes. Subsequently, RIPK1 and RIPK3 undergo phosphorylation and activation [82]. Activated RIPK3 acts on the mixed-lineage kinase domain like protein (MLKL) to phosphorylate, oligomerize, and migrate from the cytoplasm to the cell membrane to form open pores, leading to necrosis [85]. Using a mouse model of MIRI, it has been demonstrated that the levels of RIPK1, RIPK3, and MLKL are increased in the myocardial tissue following reperfusion [86]. Nevertheless, it is crucial to consider the duration of reperfusion and the species-specific vulnerability to cellular death when assessing necroptosis [86]. A study revealed that RIPK3 is also involved in ERS and intracellular Ca^2+^ overload, thereby exacerbating MIRI [87]. The understanding of RIP3-mediated necroptosis and its relationship with apoptosis and other cell death pathways in MIRI remains largely unexplored, with only a few studies addressing this topic. Caspase-8, the pivotal molecule in the process of programmed cell death, can cleave RIPK1, thereby impeding the initiation of necroptosis [82]; as such, caspase-8 represents a connection between apoptosis and necroptosis (Figure 6).

An additional pathway that appears to be involved in myocardial necroptosis is that of phosphoglycerate mutase 5 (PGAM5), which likely acts through mitochondrial fragmentation during MIRI: according to Wang et al., necroptosis can be inhibited through the degradation of PGAM5, which involves the upstream activation of AMP-activated protein kinase (AMPK) and the subsequent stabilization of Kelch-like ECH-associated protein 1 (Keap1) [88]. The molecular mechanisms of necroptosis are still partially unknown; however, in light of the aforementioned pathways and their potential as therapeutic targets in MIRI, it is considered important to further investigate this peculiar form of cell death.

## 6. MIRI and Autophagy

Autophagy is a homeostatic mechanism characterized by a lysosome-dependent degradation [89,90], which is activated by the cells as a response to stress situations, like starvation, or other environment changes [91,92]. It is possible to summarize the well-regulated autophagy process in four principal steps that include the following: (1) the induction of autophagosome formation, (2) the autophagosome formation, (3) the fusion between autophagosome and lysosome, and (4) the degradation of lysosome and its content [92,93,94]. In MIRI, autophagy could be defined as a double edge sword, because it has a cardioprotective effect during ischemia, promoting cell survival and limiting harmful consequences, but its uncontrolled increase during reperfusion becomes detrimental for cardiomyocytes [95,96]. During MIRI, the activation of Beclin 1 is essential to initiate autophagy [89], since this molecular regulator promotes the formation of autophagic lysosomes [92]. In 2007, Matsui et colleagues conducted a preclinical study on mice, concluding that the AMP-activated protein kinase (AMPK) pathway played a regulatory role in autophagy only during myocardial ischemia, and not also in the reperfusion phase, when Beclin-1 is the main molecular autophagic protein [97]. The modulation of autophagy depends on the interaction between Beclin-1 and the Bcl-2 protein, a human apoptotic regulator (anti-apoptotic) which inhibits Beclin-1 when the two molecules are linked, repressing autophagy [98]. However, during MIRI, ROS generation may cause Bcl-2 phosphorylation, promoting the dissolution of the molecular complex with Beclin-1, whose accumulation activates an uncontrolled autophagy [92,99].

On the other hand, during MIRI, the TLR4/NF-kB pathway could downregulate autophagy through the reduced expression of Beclin-1 [100]. Furthermore, another intracellular pathway probably involved in the regulation of cardiomyocyte autophagy during MIRI is the phosphoinositide 3 kinase (PI3K)/Akt/mammalian (or mechanistic) target of the rapamycin (mTOR) pathway, as demonstrated in 2020 by Qiu et al., whose results showed that downregulation of P300/CBP-associated factor (PCAF) inhibited autophagy through activation of PI3K/Akt/mTOR signaling pathway in vitro and in rat models [101]. The same inhibitory role on autophagy by the PI3K/Akt/mTOR pathway was already shown in the study by Jie et al., which was conducted on MIRI models in rats [96]. Another confirmation in the same direction was given by Chen et al., who demonstrated that hyperbaric oxygen treatment could protect against MIRI via the inhibition of inflammation and the modulation of autophagy mediated by upregulation of Akt and mTOR, and decrease in Beclin-1 and tubule-associated protein 1 light chain 3 beta (LC3B), which is another central marker in autophagy [90].

**Figure 7:**
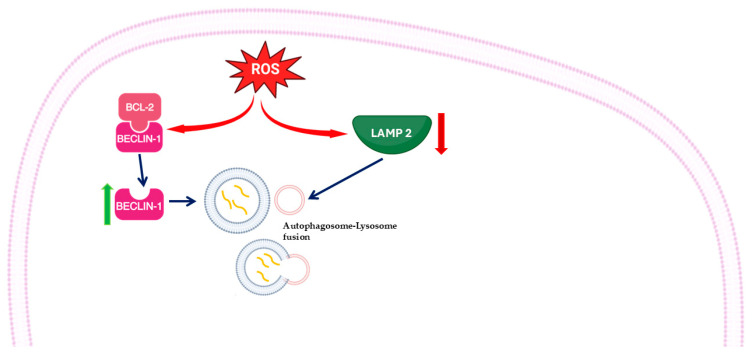
Schematic representation of autophagy’s activation during myocardial ischemia/reperfusion injury (MIRI). Increased reactive oxygen species (ROS) levels can disrupt the BCL2–Beclin-1 complex, freeing Beclin-1 to promote the formation of autophagosomes. ROS also cause a decrease in lysosome-associated membrane protein 2 (LAMP2) expression. Reduction in LAMP2 limits lysosomal degradation capacity, leading to defective autophagic flux and contributing to cellular damage under oxidative stress.

One of the mechanisms that sustains autophagy during MIRI is related to the impaired autophagic efflux that develops due to the interaction of other molecules involved in MIRI with the classical mechanisms that regulate the process under physiological conditions [102]. ROS, whose accumulation during reperfusion has been previously covered, can interact with the expression of LAMP 2 and BECN-1. LAMP 2 is required for phagosome–lysosome fusion, while BECN-1 is able to interfere with this process (Figure 7). The accumulation of ROS causes lower expression of LAMP 2 and overexpression of BECN-1; this causes accumulation of autophagosomes and consequently of damaged cellular components. An impaired autophagy flux was confirmed in other studies which suggested that MIRI may lead to the accumulation of autophagosomes [103]. This accumulation, in turn, enhances inflammation and supports the production of ROS and increased mitochondrial damage [104].

**Figure 8:**
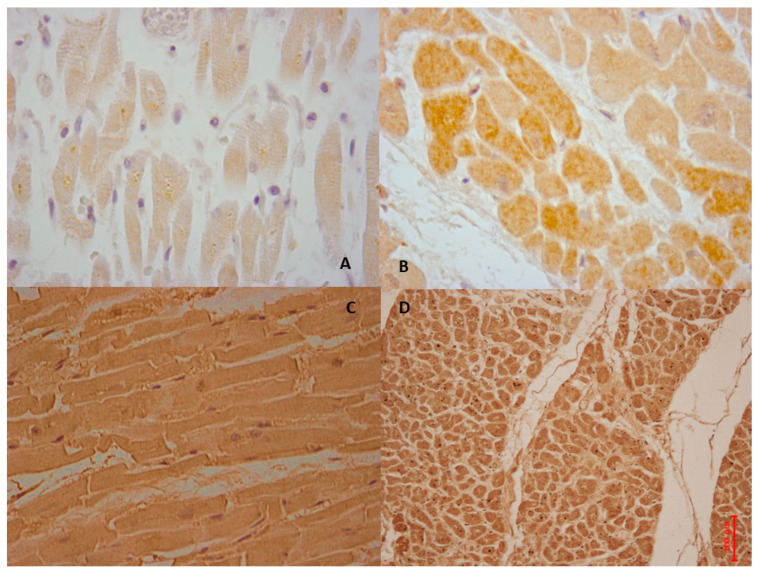
The case of a 59-year-old woman who died due to AMI (**A**). Intermediate positive reaction of BCL-2 antibody in ventricular myocytes (brown reaction). (**B**) BAX labeled myocardial cells with intensive positive (in brown) (**C**,**D**). Intense positive immunohistochemical reaction with antibody anti-Beclin-1 (**C**), and anti-ATF6 (**D**).

Apoptotic and autophagy markers are highly expressed in myocardial ischemia/reperfusion injury, as shown in Figure 8, referring to a personal observation involving a 59-year-old obese woman with a Body Mass Index (BMI) of 44. While hospitalized and awaiting bariatric surgery, she suddenly experienced chest pain and shortness of breath. A STEMI was diagnosed, and she immediately underwent PCI. Although her symptoms initially improved, six hours later, she suffered a fatal episode of VF that was unresponsive to cardiopulmonary resuscitation.

## 7. MIRI and Pyroptosis

Pyroptosis is a form of lytic, programmed cell death dependent on caspase-1, firstly observed in *Salmonella typhimurium* in 2001 [105]. Typically, this process induces formation of pores in the plasma membrane with the release of cell content [106]. This mechanism can be activated through different pathways. The canonical one is dependent on caspase-1, with the essential contribution of the aforementioned inflammasome; the non-canonical one involves caspase-4/5 and 11, activated by lipopolysaccharide (LPS) [107]. Both pathways cause the release of IL-1β and IL-18. GSDMD (Gasdermin D) is the executive protein of pyroptosis, caspase-1 cleaves GSDMD to form N- and C-terminal aminopeptide, and the GSDMD-N can in turn interact with the inner cell membrane; this interaction causes the formation of pores and unregulated leakage of cellular material, which is typical of pyroptosis [106]; GSDMD-C inhibits necrosis [108].

In 2020, Shen et al. demonstrated that in vivo mice subjected to myocardial ischemia and reperfusion had higher levels of NLRP3, ASC, caspase-1, GSDMD, and IL-1β than the sham group; these values were even higher in hyperuricemia mice. Uric acid interacts with the inflammasome, promoting its activation and contributing to ROS accumulation [20]. Later, in 2022, Ye et al. showed that GSDMD participated in MIRI, demonstrating that mice GSDMD-/- subjected to R/I reported a decrease in pro-inflammatory cytokine levels and a reduced infarct zone than WT [109].

In 2023, Zhang et al. studied the effects of cellular FLICE-like inhibitory protein L (cFLIP_L_) on the NLRP3/caspase-1/GSDMD (Gasdermin D) pathway and demonstrated its role in attenuating inflammation and modulating pyroptosis. This study showed that overexpression of cFLIP_L_ decreases the expression of NLRP3 and significantly inhibits the production of the mature caspase-1, resulting in the reduction in IL-1β and IL-18 levels, potentially acting as a natural inhibitor in inflammasome activation [108].

Pyroptosis can also be regulated by calcium overload through calpains. Calpains are calcium-dependent nonlysosomal cysteine proteinases; their activation has been previously demonstrated during MIRI following increased intracellular calcium concentration [110,111]. In 2019, Yue et al. investigated the involvement of calpain in the pyroptotic process using mouse models in vivo and in vitro; the authors showed that calpain can interact with the NLRP3/ASC/caspase-1 axis and participate in the induction of pyroptosis [112].

The markers of pyroptosis are involved in MIRI, in particular calpain 1 and 2. Calpains are proteins that depend on calcium to work and are found in the cells’ fluid in an inactive state. They can be activated in various situations and play a crucial role in activating different proteins. These include growth factor receptors, proteins that comprise the cell’s structure, proteins that interact with microtubules, and those located in mitochondria. Calpains play a crucial role in regulating the cell cycle, programmed cell death (apoptosis), and cell development [112,113,114].

The process of activating calpains starts when calcium levels inside the cell increase. Changes in calpain’s shape enable it to move to the cell membrane, where specific fats help lower the calcium levels required for activation or position calpain near calcium channels, leading to protein activation. Many heart problems are linked to imbalanced calcium levels, particularly during heart damage caused by a lack of blood flow (ischemia) and subsequent restoration of blood flow (reperfusion). Studies of heart tissues have shown that ischemia/reperfusion leads to higher levels of calcium inside the cells. When blood supply to the heart is reduced, sodium and calcium ions accumulate, causing a drop in pH and resulting in tissue acidity. When blood flow returns, there are quick changes in ion movements, which affect how ions are exchanged in the body. The combined harmful effects of ischemia and reperfusion result in excessive calcium accumulation within cells [115,116,117,118].

**Figure 9:**
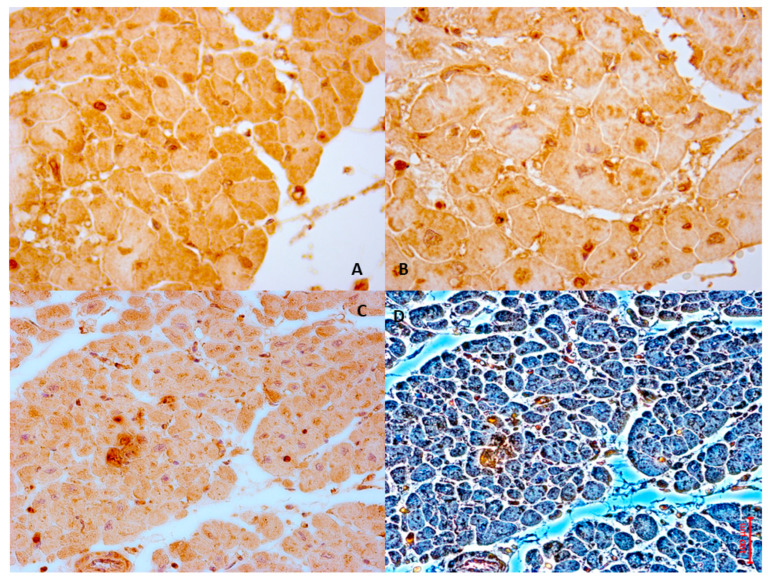
The case of a 78-year-old man who died after a post-surgery STEMI treated with PPCI. (**A**,**B**). Strong immunolocalization of calpain-1 in ventricular myocytes (brown reaction). (**C**) Intense positive immunohistochemical reaction with antibody anti-calpain-1 in bright field, and the same field with phase contrast (**D**).

In Figure 9, we show a personal observation of a 78-year-old man who underwent PPCI for STEMI three months prior to having ankle prosthesis surgery. Forty minutes after the surgery, he experienced the onset of symptoms consistent with STEMI. He was admitted to the coronary care unit, where a coronary angiogram revealed an occluded descending coronary artery. PPCI was performed promptly. However, two hours later, he developed VF and cardiac arrest, which was unresponsive to cardiopulmonary resuscitation.

**Figure 10:**
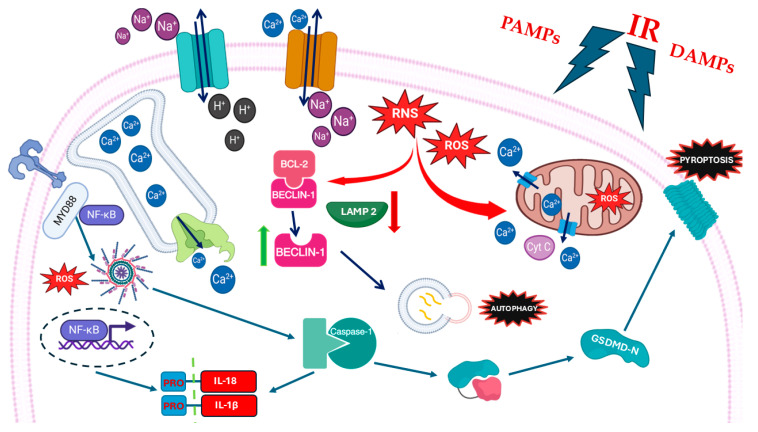
Schematic representation of pathways activated by pathogen-associated molecular patterns (PAMPs) and damage-associated molecular patterns (DAMPs) during myocardial ischemia/reperfusion injury (MIRI). Recognition of these signals triggers downstream responses, including pyroptosis, autophagy, and inflammasome activation, contributing to cardiomyocyte injury and inflammation.

In summary, pyroptosis is a highly complex process, and the multiple pathways discussed above are involved in MIRI (Figure 10).

## 8. Focus on Novel Therapeutic Strategies: Certainties and Hopes

### 8.1. Inflammation as an Actionable Target for the Secondary Prevention of Cardiovascular Events

In recent years, multiple clinical trials investigated anti-inflammatory therapeutic strategies for the secondary prevention of cardiovascular events in patients with coronary artery disease, primarily favoring plaque stabilization. Starting from 2011, the Canakinumab Anti-inflammatory Thrombosis Outcome Study (CANTOS) [119] tried to understand whether specifically targeting the IL-1β axis (Canakinumab is a human monoclonal antibody against IL-1β) might lead to a reduction in secondary cardiovascular events, as finally demonstrated when the study results were made publicly available in 2017 [11]. The use of Canakinumab 150 mg every 3 months is currently limited in clinical practice, because the reduction in atherothrombotic events is counterbalanced by an increased risk of infections, and also because of the high cost. More recently, a further and astonishing confirmation that modulating the inflammatory cascade may result in a reduction in atherothrombotic events has come with the approval of Colchicine (0.5 mg daily) by the United States Food and Drug Administration (FDA) in July 2023 as the first anti-inflammatory drug for secondary prevention of cardiovascular events. Furthermore, Colchicine is also now featured in the 2023 European Society of Cardiology guidelines for the treatment of acute coronary syndromes [120], which report a class IIb level of evidence A recommendation for the use of the drug for the long-term management of ACS patients if other risk factors are insufficiently controlled or in case of recurrent events under optimal therapy. This is the result of ten years of research on Colchicine (a natural alkaloid extracted from the *Colchicum autumnale* plant), the anti-inflammatory effects of which were already known, and which was already used for treating inflammatory diseases (gouty arthritis, pericarditis, and others). Colchicine was tested in three large randomized controlled clinical trials enrolling patients with coronary artery disease, LoDoCo, COLCOT, and LoDoCo2 [121,122,123], which assessed the drug’s beneficial properties in terms of reductions in atherothrombotic events. The successful outcome in secondary cardiovascular prevention is certainly due to the mechanisms of inhibition of microtubule formation and cellular trafficking, but moreover to the inhibitory action on the NLRP3 inflammasome and hence on the release of IL-1β and IL-18, as well as of other pro-inflammatory cytokines, such as IL-6 (surface expression and downstream pathway) [124,125]. Many other drugs targeting the immune system are being studied to find novel therapies in patients with AMI. In this context, Anakinra (a recombinant IL-1 receptor (IL-1R) antagonist) appears to be promising, with a significant reduction in inflammatory markers (i.e., hsCRP) and risk of heart failure (HF) in the VCUART 3 trial [126,127,128]. Ziltivekimab (a fully human monoclonal antibody against IL-6) is another possible agent for the secondary prevention of atherosclerotic cardiovascular disease, as demonstrated in the RESCUE trial (phase 2) [129], and more recently in ZEUS [130,131].Tocilizumab (a monoclonal antibody against IL-6R) was investigated for secondary prevention of AMI, both NSTEMI [132] and STEMI [133], and has shown interesting results consisting in lower myocardial injury (lower levels of troponin T), with a reduction in inflammatory markers (i.e., hsCRP). Another drug lowering IL-6 levels, Hydroxychloroquine, seemed to reduce cardiovascular endpoints after myocardial infarction [134]. On the other hand, immune system mediators (i.e., low dose IL-2) were also evaluated as drugs for secondary cardiovascular prevention [135,136].

Although all these drugs modulating the inflammatory response were evaluated for the reduction in cardiovascular events, whether the beneficial effect may have also depended on an amelioration of the I/R injury besides atherosclerotic plaque stabilization is currently unknown and deserves further study. In fact, Colchicine and Tocilizumab were recently investigated in two randomized, double-blind, placebo-controlled trials, respectively, COVERT-MI, 2021 [137], and ASSAIL-MI, 2021 [133], as cardioprotective adjunctive therapies in patients with STEMI undergoing PPCI, resulting in no significant differences in final IS for Colchicine, but in an amelioration of the myocardial salvage index in STEMI patients treated with Tocilizumab.

### 8.2. MIRI Modulation in the Clinical Arena

A huge miscellaneous pool of agents (not only drugs) is being investigated as potential new cardioprotective therapies specifically for the mitigation of MIRI, but none has entered routine clinical practice yet. In fact, starting from promising results in experimental animal studies, multiple heterogeneous approaches were recently tested in clinical trials to confirm the potential cardioprotective effects against MIRI. In these studies, the population was often characterized by patients presenting with STEMI and undergoing PPCI, with a periprocedural administration of the strategy (physical or pharmacological one) under study, before or immediately after the reperfusion. The most common primary outcome in almost all studies was the reduction in IS as measured by late gadolinium enhancement (LGE) on cardiac magnetic resonance (CMR). Serum inflammatory (IL-1, IL-6, TNFα, …) and/or cardiac necrosis (Tn, CK-MB) biomarkers were also often measured, as well as the incidence of major cardiovascular events (MACEs). It is important to note that most of these clinical trials failed to show clear results in terms of clinical efficacy. The explanation for this failure is obviously multifactorial, and probably due to the difficulties in the following items/aspects: design of the clinical trials; identification of correct therapeutic window for drug administration; and elimination of redundancy with other therapies (the P2Y inhibitor antiplatelet therapy that it is normally administered before PPCI) to be sure that possible beneficial effects are linked to the compound under study and not to confounding elements.

All these potential cardioprotective strategies aiming to reduce MIRI, even if very heterogeneous and acting at the level of different intracellular molecules and signaling pathways, mainly converge on the following: (1) reduction in oxidative stress (both by the reduction in formation of ROS and RNS, and by an increase in their removal), (2) blunting of inflammation, and (3) resolution of mitochondrial dysfunction, acting on the inhibition of mPTP (mitochondrial permeability transition pore) opening, on the mitochondrial respiratory chain, and on the induction of autophagy by damaged mitochondria (mytophagy), which are all overlapping mechanisms. Individual approaches are briefly discussed in the following text.

First of all, myocardial conditioning (pre-, post-, and remote ischemic conditioning) has shown promising results in experimental animal studies (probably due to multifactorial mechanisms causing a final reduction in ROS and RNS production, through changes in NOS expression and intracellular pH), although not confirmed in the clinical setting, as demonstrated by two recent large randomized control trials (RCTs) which failed to show improved clinical outcomes, respectively, through ischemic postconditioning (DANAMI-3, 2017) [138] and remote ischemic conditioning (CONDI-2/ERIC-PPCI, 2019) [139]. Actually, regarding the latter mechanism, a prospective single-center randomized trial (RIC-STEMI, 2018) showed positive clinical results [140], which were confirmed in a recent post hoc analysis [141].

Another non-pharmacological approach is represented by intracoronary administration of hyperbaric super-saturated oxygen (SSO2) immediately after PPCI, which may act by improving the endothelial structure and function after AMI, through an increase in oxygen supply and of the production of endogenous ROS scavengers, as well as a reduction in myocyte apoptosis [142]. These potentially beneficial mechanisms translated into a reduction in IS, as shown in a recent multicenter clinical study (IC-HOT, 2019) [143], following two important previous randomized trials on “The Acute Myocardial Infarction with Hyperoxaemic Therapy” (AMIHOT I-II, 2007–2009) [144,145], and nowadays confirmed in recent comprehensive reviews.

Therapeutic hypothermia is another promising non-pharmacological cardioprotective strategy, whose beneficial role seems to be due to a reduction in hypercontractile state of myofibrils, and preservation of mitochondrial bioenergetics [146]. Other non-pharmacological therapies against MIRI are currently being studied in clinical trials such as extracorporeal cardiac shock wave therapy intervention [147].

Regarding “pure” pharmacotherapy, the “Cyclosporine and Prognosis in AMI Patients” (CIRCUS, 2015) trial studied Cyclosporine A, an inhibitor of mPTP (actually not a blocker, but an increaser of the opening threshold), but without successful clinical effects [148]. A recent phase II clinical trial by de Konig et al. [149] investigated the potential protective effect of Sodium Thiosulfate (STS), an antioxidant and H_2_S donor, against ischemia/reperfusion injury in patients presenting with STEMI, but the results showed neither an infarct size reduction nor clinical benefits. Another field of investigation concerned the use of intravenous (IV) beta-blocker administration before PPCI, as assessed in an interesting RCT by Ibanez et al. [150], The Effect of Metoprolol in Cardioprotection During an Acute Myocardial Infarction (METOCARD-CNIC, 2013) trial. This study demonstrated the cardioprotective beneficial effects of intravenous metoprolol administration before reperfusion in patients presenting with STEMI, as confirmed by a recent further electrocardiographic study [151]. The pathophysiological mechanism is probably related to the reduced activation of different matrix metalloproteinases (MMPs) in cardiomyocytes, and, therefore, to a reduced cleavage of sarcomeric proteins, with an amelioration of the cardiac contractile dysfunction related to MIRI [152]. Similar results were obtained in a RCT testing Landiolol, suggesting that this effect may be shared by different beta-blockers [153].

Recently, a multicenter, prospective, randomized, double-blind phase IV clinical trial by Díaz -Munoz R. et al. demonstrated beneficial cardioprotective effects of a hybrid ATP-sensitive potassium channel opening agent, Nicorandil, when intravenously administrated before PCI in patients with STEMI (CHANGE, 2022) [154].

Other promising therapeutic strategies for MIRI are represented by the inhibition of Class-I histone deacetylases (HDACs), such as Mocetinostat (MGCD0103, MOCE), which lead to stimulation of autophagy [155,156]. Oxidized mtDNA and pyroptosis are other targets of cardioprotection. The repair and/or reduced formation of damaged mtDNA may be achieved by reducing oxidative stress, thus supporting the interconnection of the various pathophysiological mechanisms implicated in MIRI. Furthermore, consistent with the signaling pathways described in the previous section, NLRP3 inflammasome, TLR-9, and caspase-1 may be targeted with inhibitory compounds, leading to a reduction in both the synthesis of cytotoxic cytokines (IL-1 and Il-18), and pyroptosis, as well as to greater cell survival. Among these substances, it is worth mentioning Tongxinluo, a traditional Chinese medicine with promising in vitro results, mediated by a reduction in pyroptosis in endothelial cells, on the activation of caspase-1, and on the release of inflammatory cytokines, and with recent clinical beneficial effects demonstrated in a just-published randomized, double-blind, placebo-controlled Chinese phase IV clinical trial (CTS-AMI, 2023) [157].

Moreover, many compounds routinely used in different treatment/clinical settings were tested as cardioprotective agents against MIRI. For example, the biguanide Metformin, a well-known oral antidiabetic drug, was evaluated against placebo in non-diabetic STEMI patients after PCI (GIPS-III RCT, 2017) [158].

Even though the RCT’s results were negative for significant clinical benefits, Metformin remains an interesting compound with potential significant cardioprotective effects, considering the multiple mechanisms of action against MIRI (activation of the AMPK pathway, activation of several kinases of the RISK, attenuation of mitochondrial dysfunction, decrease in myocardial oxidative stress, reduction in apoptosis, etc.) [159].

Another example is represented by vitamin C, which was associated with reduced cardiac enzyme levels in patients with STEMI undergoing PCI or cardiac surgery, preserved endothelial function, and lower production of harmful ROS [160]. High-dose intravenous vitamin C seems to also be effective during I/R after a cardiac arrest, as demonstrated in a phase II clinical trial [161].

Another small clinical trial showed that Glutathione, a water-soluble tripeptide with a potent oxidant scavenging activity, when administrated before PCI in STEMI patients, may reduce the deleterious effects of H_2_O_2_ generation on the myocardium [162].

Some of the heterogeneity in observed clinical trials may depend on the influence of metabolic comorbidities, especially hypercholesterolemia and hyperglycemia, which were reported to attenuate the cardioprotective effects of myocardial conditioning in an experimental study [163]. Whether pharmacologic modulation of hypercholesterolemia and hyperglycemia may specifically improve MIRI is an open question, the verification of which in the clinical arena is difficult to achieve, due to the contemporary implication of these risk factors on both atherothrombosis and MIRI.

The main characteristics of the most recent/relevant clinical trials investigating several pharmacological and non-pharmacological approaches acting on MIRI are summarized in Table 1.

### 8.3. What Hopes?

Analyzing the literature and the characteristics of individual trials confirms the difficulty of translating what appears effective in in vitro or in animal models into clinical practice. It becomes evident that selectively targeting MIRI in the context of highly complex and multifactorial injuries is a considerable challenge. In most studies, the primary endpoint is infarct size; however, the etiology of myocardial damage cannot be attributed solely to MIRI, but rather—and primarily—to the hypoxic injury. The choice of endpoint to be evaluated, as well as the timing and dosage of potential drugs and/or therapeutic interventions, may represent significant limitations to the success of clinical trials. Therefore, the authors hope to learn from the numerous studies conducted thus far and to refine future trial designs and therapeutic strategies without neglecting fundamental preclinical research, which remains an indispensable driving force.

## 9. Conclusions

The present work confirms that MIRI is a fearful condition that can seriously worsen the outcome of myocardial infarction. Although effective cardioprotective therapies applicable in clinical practice are not available yet, the authors have demonstrated that the goal of understanding the complex and multifactorial pathophysiology of MIRI remains essential, both for clinical and forensic purposes. In fact, only the increasing knowledge of the underlying inflammatory mechanisms and the tissue changes triggered by the multiple molecular/cellular pathways can guarantee the creation of well-designed clinical trials, moving from animal studies, with the final aim to find successful therapeutic targets. Furthermore, the histological and immunohistochemical case series presented from the review authors’ institution has an exceptional value because of its exclusivity in the literature and because it underlines the importance for forensic pathologists to be aware of the existence and pathophysiology of MIRI, both to contribute to the tissue characterization of this damage and to recognize the potential deaths related to MIRI, despite early and adequate reperfusion treatment, in malpractice claims.

In conclusion, there is still a great deal of study which pathologists, cardiologists, biologists, and other researchers have to continue on this fundamental topic, focusing on the centrality of the tissue pathology characterization of MIRI.

## Figures and Tables

**Table 1:** Characteristics of the clinical trials investigating several pharmacological and non- pharmacological approaches acting on MIRI.

Study Name/ClinicalTrials.gov Identifier	Intervention/ Treatment	Intervention Target	Study Design	Timing of Administration of the Treatment	No. (Drug vs. Control) Patient Cohort	Primary Study End Point	Main Outcomes	Main Author, Date, Journal [Reference]
**PULSE-MI** (Pulse Glucocorticoid Therapy in Patients With ST-Segment Elevation Myocardial Infarction)/**NCT05462730**	**Methylprednisolone** vs. placebo	**Inflammation** (genomic and non-genomic effect of glucocorticoids, decreasing vascular inflammation through membrane stabilization, and increasing contractility of the vascular smooth muscle cells)	Randomized, double-blind, placebo-controlled	In the pre-hospital setting, bolus infusion of 250 mg (1 × 4 mL) over a period of 5 min	530 patients (378 patients completing the CMR at 3 m) with STEMI undergoing PPCI	Reduction in IS (in % of LV mass) as assessed by LGE on CMR at 3 months	No significant differences	Madsen JM, 2024, JAMA Cardiol. [164]
**CTS/AMI** (China Tongxinluo Study for Myocardial Protection in Patients With Acute Myocardial Infarction)/**NCT03792035**	**Tongxinluo** vs. placebo	**Inflammation pyroptosis** (reduction in pyroptosis of endothelial cells, in the activation of caspase-1, and in the release of inflammatory cytokines)	Randomized, double-blind, placebo-controlled	After randomization (loading dose of 2.08 g), followed by the maintenance dose of 1.04 g, t.i.d.)	3777 patients (Tongxinluo: 1889 vs. 1888) with STEMI within 24 h of symptom onset	MACE at 30 d and at 1 y	Decrease in MACE both at 30 d and at 1 y in the Tongxinluo group compared with the placebo group	Yang Y, 2023, JAMA [157]
**GIPS-IV** (Groningen Intervention Study for the Preservation of Cardiac Function with STS after STEMI)/**NCT02899364**	**Sodium Thiosulfate (STS)** vs. placebo	**Oxidative stress, apoptosis, inflammation, mitochondrial and microvascular function** (antioxidant and H_2_S donor)	Randomized, double-blind, placebo-controlled	Before and during PPCI, bolus infusion of 12.5 mg over 20–30 min, followed by 12.5 mg at 6 h post-PPCI	373 patients (226, 116 vs. 110, completing the CMR at 4 m) with first-presentation STEMI within 12 h symptom onset, undergoing PPCI	Reduction in IS (in % of LV mass) as assessed by LGE on CMR at 4 m	No significant differences	de Koning MS, 2023, JACC Basic Transl Sci. [149]
Substudy of **ASSAIL-MI** (ASSessing the Effect of Anti-IL-6 Treatment in Myocardial Infarction: The ASSAIL-MI Trial)/**NCT03004703**	**Tocilizumab** vs. placebo	**Inflammation** (antagonism of IL-6 receptor by a recombinant humanized monoclonal antibody)	Randomized, double-blind, placebo-controlled	Before PPCI, bolus infusion of 280 mg (14 mL) over a period of 1 h	199 adult STEMI patients (101 vs. 98), within 6 h symptom onset before PPCI	Reduction in IS (in % of LV mass) as assessed by LGE on CMR at 3–7 d and at 6 m	Tocilizumab increased the concentrations of most cytokines (IL-6, IL-8 and IL-1ra) in the acute phase compared with placebo, but decreased neutrophils/CRP (markers of MI). In the ASSAIL-MI, Tocilizumab increased the MSI	Woxholt S, 2025, Int J Cardiol. [165]; Brock K, 2021, JACC. [133]
Substudy of **RIC-STEMI** (Remote Ischemic Conditioning in ST-elevation Myocardial Infarction)/**NCT02313961**	**RIC** (3 cycles of manual inflation of a blood pressure cuff placed on the left lower limb to 200 mmHg for 5 min and then deflation to 0 mmHg for another 5 min)	Cardioprotection of brief cycles of non-lethal ischemia and reperfusion applied to a distant organ before, during or after a long period of myocardial ischemia	Randomized, open label	At the time of PPCI	448 STEMI patients undergoing PPCI without (217) or with RIC (231); patients’ division also according to the time of PPCI (216 night-morning vs. 232 afternoon)	Composite: cardiac death or HF hospitalization on a minimum follow-up of 12 m	At 2.1 y of follow-up, improvement in outcomes in patients undergoing RIC as an adjunct to PPCI. The substudy showed a daytime variation in clinical results suggesting that the afternoon period enhances the cardioprotection induced by RIC	Pires CM, 2023, Heart Vessels [141]; Gaspar A, 2018, Basic Res Cardiol. [140]
**PENTOS-PCI**	**Pentoxifylline** vs. placebo	**Inflammation, oxidative stress** (methylxanthine derivative with known anti-inflammatory, antioxidant, vasodilator, and rheological properties)	Randomized, double-blind, placebo-controlled	During PPCI, 100 mg (bolus infusion of 50 mg followed by 50 mg in 30 min)	161 (80 vs. 81) adult patients with STEMI eligible for PPCI	PCI’s success rate as measured by TIMI flow grade	No significant difference	Kakavand H, 2023, Naunyn Schmiedebergs Arch Pharmacol. [166]
**RESTORE** (Randomized Evaluation of Shenfu Injection to Reduce Myocardial Injury)/**NCT04493840**	**Shenfu** vs. placebo	**Inflammation, oxidative stress, apoptosis, calcium overload** (scavenging free radicals, inhibiting inflammatory mediators, suppressing cell apoptosis, and inhibiting Ca 2+ overload)	Randomized, double-blind, placebo-controlled	Within 30 min before PPCI infusion of 80 mg (+70 mL 5% glucose injection) followed by once a day until 5 days after PPCI	326 adult patients with first-time anterior STEMI undergoing PPCI within 12 h of symptom onset	Reduction in IS (in % of LV mass) as assessed by LGE on CMR at 5 ± 2 d	-	Wang X, 2023, Am Heart J. [167]
**SALVAGE-MI** (UpStreAm doxycycline in ST-eLeVation myocArdial infarction: targetinG infarct hEaling and ModulatIon)	**Doxycycline**	**Inflammation, oxidative stress, apoptosis, and especially MMPs** (inhibitory properties)	Randomized, double-blind, placebo-controlled	Before PPCI bolus infusion of 100 mg over 5 min followed by an oral dose of 100 mg (b.i.d.) for 7 d	103 patients (50 vs. 53) with first-presentation STEMI within 12 h symptom onset, TIMI flow 0–1 undergoing PPCI	Reduction in final IS adjusted for area-at-risk (fIS/AAR) measured on 2 CMR (the first one, 1–2 w post-PPCI and the second one, between 3 and 6 m)	No significant difference	Noaman S, 2023, Eur Heart J Acute Cardiovasc Care [168]
**CHANGE** (China-Administration of Nicorandil Group)/**NCT 03445728**	**Nicorandil** vs. placebo	**Microvascular function** (a hybrid ATP-sensitive potassium channel opening agent)	Randomized, double-blind, placebo-controlled	Before PPCI bolus infusion of 12 mg and then after PPCI, continuous infusion of 6 mg/h. up to 24 h	238 STEMI patients (120 vs. 118) undergoing PPCI	Reduction in IS (in % of LV mass) as assessed by LGE on CMR at 7 d and at 6 m post-PPCI	Nicorandil lead to improved myocardial perfusion grade, increased left ventricular ejection fraction, and reduced myocardial IS	Qian G, 2022, J Am Heart Assoc. [154]
**GUARD** (Gradual Versus Abrupt Reperfusion During PPCI)/**NCT 02732080**	**Pressure-controlled reperfusion with delayed stenting (PCRDS) vs. standard PCI with immediate stenting**	**Microvascular integrity**	Randomized, open label	After PCI	30 adult STEMI patients undergoing PPCI with achieved TIMI flow 3	Coronary zero flow pressure (Pzf) at the end of the 1 h intracoronary hemodynamic monitorization	PCRDS lead to better-preserved coronary microvascular integrity and smaller myocardial IS	Sezer M, 2022, J Am Heart Assoc. [169]
**A Study of Acute Myocardial Infarction Using FDY-530/NCT03470441**	**FDY-5301** vs. placebo	**Oxidative stress** (catalytic anti-peroxidant agent)	Randomized, double-blind, placebo-controlled	Before PPCI, infusion of 0.5, 1.0 or 2.0 mg/kg or placebo	120 STEMI patients presenting within 12 h symptom onset undergoing PPCI	Incidence of cardiac arrhythmias of interest from 48 h to 14 d post-PPCI	No significant difference in the primary endpoint, but difference (without statistical significance) in reduction in IS and improvement of LVEF. Moreover, significant reduction in the levels of MPO, MMP2 and NTproBNP after PPCI	Adlam D, 2022, Int J Cardiol. [170]
**GSH/EudraNCT2014-004486-25**	**Glutathione** vs. placebo	**Oxidative stress, inflammation** (modulation of innate immune cell recruitment and reduction in endothelial damage by reducing NOX2-mediated inflammatory process, and the deleterious effects of H2O2 generation on myocardium)	Randomized, double-blind, placebo-controlled	Before PPCI infusion of 2500 mg/25 mL over 10 min followed by infusion at the same dose at 24, 48 and 72 h	100 adult STEMI patients undergoing PPCI	Reduction in plasma levels of oxidants and inflammatory markers (NOX2, TNFa, NO, H2O2) at 2 and 5 d	Early and prolonged glutathione infusion seems able to protect vital myocardial components and endothelial cell function against harmful pro-oxidant and inflammatory environments	Tanzilli G, 2021, JAHA [162]
**VITaCCA** (Vitamin C in Post-cardiac Arrest)/**NCT03509662**	**Vitamin C**	**Oxidative stress, endothelial function** (scavenger of free radicals, reduction in the production of ROS)	Randomized, double-blind, placebo-controlled	Infusion of 1.5 gr or 5 gr b.i.d. (3 gr/d or 10 gr/d) for 4 d	270 (90 vs. 90 vs. 90) comatose patients suffering an out-of-hospital cardiac arrest	ΔSOFA (the difference between SOFA admission and SOFA at 96 h)	-	Rozemeijer S, 2021, Trials [161]
**METOCARD-CNIC** (Effect of METOprolol in CARDioproteCtioN During an Acute Myocardial InfarCtion)/**NCT01311700**	**Metoprolol (beta-blocker)**	**MMPs** (reduction in sarcomeric proteins cleavage with an amelioration of the cardiac contractile dysfunction)	Randomized, controlled parallel-group, observer-blinded	Before PPCI, intravenous infusion of up to three 5 mg i.v. dosages (2 min apart). 12–24 h post-reperfusion, oral treatment (25–100 mg/12 h), for all patients	270 (131 vs. 139) anterior STEMI patients	Reduction in IS evaluated primarily by area of delayed enhancement on CMR at 5–7 d after PPCI and time-dependent progression of ischemic injury assessed by serial ECG	Early intravenous metoprolol before PPCI reduces IS and increases LVEF with no excess of adverse events during the first 24 h after STEMI. Moreover, ECG markers of myocardial ischemia ameliorate (ECG substudy)	Díaz -Munoz R, 2021, Basic Res Cardiol. [151]; Ibanez B, 2013, Circulation [150]
**EUROCRIPS** (Efficacy of RIPC to Reduce AKI for Patients Undergoing PCI)/**NCT02195726**	**RIC** (5 min inflations of a blood pressure cuff to 200 mmHg around the upper non dominant arm) vs. sham-procedure	**RISK pathway** (EV-naive led to STAT-3 phosphorylation, while EV-RIPC to Erk-1/2 activation)	Randomized, double-blind, sham-controlled	Before PPCI, 4 cycles of RIC and then collection of serum EVs (extracellular vesicles)	30 ACS patients undergoing PPCI	Incidence of acute kidney injury and of periprocedural MI at 24–48 h after PPCI	TnT is enriched in circulating EV from ACS patients; EV-naïve have a cardioprotective activity through SAFE pathways, lacking in EV-RIPC	D’Ascenzo F, 2021, Pharmacol Res. [171]
**The Acute and Chronic Effects of Remote Ischemic Conditioning on Cardiovascular Function/NCT03984123**	**RIC** vs. sham procedure	**Oxidative stress, endothelial function**	Randomized, double-blind, sham-controlled	Within 48 h post-PPCI, one or two cycles of bilateral brachial cuff inflation	270 STEMI patients undergoing PPCI	Changes of aortic stiffness, endothelial glycocalyx integrity, oxidative stress biomarkers, miRNA expression, Nitrate-nitrite-nitric oxide plasma concentrations at baseline, 10, 25, and 45 min	RIC evokes “vascular conditioning” likely by upregulation of cardioprotective microRNAs, NOx production, and oxidative stress reduction, facilitating reverse LV remodeling	Ikonomidis I, 2021, Basic Res Cardiol. [172]
**COOL-MI** (Hypothermia as an Adjunctive Therapy to Percutaneous Intervention in Patients With Acute Myocardial Infarction)/**NCT02664194**	**Endovascular hypothermia** vs. sham procedure		Randomized, open label	Before reperfusion, 1 h or 3 h of intravascular hypothermia (1 L cold saline (1–4 °C) associated with the Proteus™ System, by cooling for at least 18 min with a target temperature of 32 °C ± 1 °C)	70 Anterior or Inferior STEMI patients undergoing PPCI	Reduction in IS (in % of LV mass) as assessed by LGE on CMR, improvement of LVEF on CMR and incidence of MACE at 30 d after STEMI	No difference in IS or LVEF at 30 d nor in MACE, but there was a higher incidence of arrhythmia and in-hospital infection in the hypothermia group, with no increase in mortality	Dallan LAP, 2021, Ther Hypothermia Temp Manag. [173]
**COVERT-MI** (COlchicine for Left VEntricular Remodeling Treatment in Acute Myocardial Infarction)/**NCT03156816**	**Colchicine** (oral) vs. placebo	**Inflammation** (inhibition of NLRP3 inflammasome, and hence on the release of IL-1β and IL-18, as well as of other pro-inflammatory cytokines, such as IL-6 (surface expression and downstream pathway))	Randomized, double-blind, placebo-controlled	At the time of revascularization (loading dose of 2 mg), followed by 0.5 mg b.i.d. for 5 d	192 adult patients (101 vs. 91), with a first STEMI (initial TIMI flow ≤1), referred for PPCI	Reduction in IS (in % of LV mass) as assessed by LGE on CMR at 5 ± 2 d	No difference in IS (at CMR) neither at 5 d nor at 3 m between Colchicine and placebo	Mewton N, 2021, Circulation [137]
**GOLD-PCI** (GLP-1 Loading During Elective Percutaneous Coronary Intervention)**/NCT02127996**	**Glucagon-like peptide 1 (GLP-1)** vs. placebo	**Cardiac and vascular myocytes’ function** (increased cGMP release, vasodilatation, and coronary flow through GLP-1R)	Randomized, double-blind, placebo-controlled	During PPCI infusion of 1.2 pmol/Kg/min	192 adult patients (101 vs. 91), with a first STEMI (initial TIMI flow ≤1), referred for PPCI	Incidence of troponin I elevation at 6 h post-PPCI and MACE up to 6 m	-	Giblett JP, 2019, Am Heart J. [174]
**MINIMIZE-STEMI** (Early Mineralocorticoid Receptor Antagonist Treatment to Reduce Myocardial Infarct Size)/**NCT01882179**	**Mineralocorticoid receptor antagonist (MRA)** vs. placebo	**Inflammation, oxidative stress** (Activity on adenosine receptor, protein kinase C, PI3-kinase, and ERK. Upregulation of phosphorylation of Akt and ERK1/2)	Randomized, double-blind, placebo-controlled	Before PPCI, infusion of potassium-canrenoate, followed by oral spironolactone 25 mg daily for 3 m (up titrated to 50 mg daily after 2 w, if possible)	67 STEMI patients presenting within 12 h and with a proximal coronary artery occlusion with TIMI flow 0	Reduction in IS (in % of LV mass) as assessed by LGE on CMR at 3 m after STEMI	No significant difference in the acute IS and final IS at 3 m, but there was an improvement in LVEF at 3 m	Bulluck H, 2019, Am Heart J [175]; Bulluck H, 2015, Clin Cardiol. [176]
**NITRITE-AMI** (Safety and Effectiveness of Intra-coronary Nitrite in Acute Myocardial Infarction)**/NCT01584453**	**Sodium nitrite** vs. placebo	**Inflammation**	Randomized, double-blind, placebo-controlled	During PPCI, an intracoronary bolus infusion of 1.8 micromol in 10 mL over 1 m	80 STEMI patients undergoing PPCI	IS measured by CK area under the curve at 48 h post PPCI and incidence of MACE at 3 y, markers of inflammation	Important reductions in neutrophil numbers and activation post-PPCI, associated with a reduction in both microvascular obstruction and IS	Jones DA, 2019, Int J Cardiol. [177]; Jones DA, 2017, Heart [178]
**Effect of N-acetylcystein in Myocardial Infarction/NCT01741207**	**N-acetylcysteine (NAC)** vs. placebo	**Oxidative stress**	Randomized, double-blind, placebo-controlled	Before PPCI, bolus infusion of 100 mg/kg and then 480 mg intracoronary, followed by 10 mg/kg/h over 12 h after PPCI	100 STEMI patients undergoing PPCI	Biomarkers of platelet activation (P selectin- CD40L-IL10- TGF-beta) after 24 h and Cardiac Necrosis Biomarkers (CKMB, troponin T) at 12 h, MACE at 30 d	NAC improved myocardial reperfusion markers and coronary blood flow, as revealed by differences in peak hs-TnT and TIMI flow grade 3 levels	Nozari Y, 2018, Am J Cardiovasc Drugs [179];
**MITOCARE**	**TRO40303** vs. placebo	**Mitochondrial function** (mPTP opening inhibitor)	Randomized, double-blind, placebo-controlled	Before PPCI, intravenous infusion and intracoronary bolus	163 STEMI patients (83 vs. 80) with chest pain within 6 h before admission for PPCI	Serum levels of pro-inflammatory cytokines (IL-1β, IL-6, IL-10, TNF), and of acute-phase proteins (hs-CRP)	No statistically significant differences	Butt N, 2017, Cardiology [180]; Atar D, 2015, Eur. Heart J [181]
**MARIA** (The Melatonin Adjunct in the Acute myocaRdial Infarction Treated With Angioplasty)/**NCT00640094**	**Melatonin** vs. placebo	**Oxidative stress** (direct free radical scavenging activities and indirect actions in stimulating antioxidant enzymes)	Randomized, double-blind, placebo-controlled	During PPCI, intravenous infusion of 12 mg over 1 h, followed by intracoronary bolus of 2 mg	146 STEMI patients presenting within 6 h of chest pain onset	Reduction in IS (in % of LV mass) as assessed by LGE on CMR at 5–7 d after reperfusion	Melatonin in patients with STEMI who presented early after symptom onset was associated with a significant reduction in the IS after PPCI	Dominguez-Rodriguez A, 2017, Am J Cardiol. [182]
**NACIAM** (N-acetylcysteine in Acute Myocardial Infarction)	**N-acetylcysteine (NAC)** vs. placebo	**Oxidative stress**	Randomized, double-blind, placebo-controlled	Intravenous high-dose (29 g over 2 d) with background low-dose nitroglycerin (7.2 mg over 2 d)	112 STEMI patients undergoing PPCI, with 75 completing CMR follow-up (37 vs. 38)	Reduction in IS (in % of LV mass) as assessed by LGE on CMR at 3 m after STEMI	High-dose intravenous NAC administered with low-dose intravenous nitroglycerin is associated with reduced IS	Pasupathy S, 2017, Circulation [183]
**EMBRACE** (Evaluation of Myocardial Effects of MTP-131 for Reducing Reperfusion Injury in Patients With Acute Coronary Events)**/NCT01572909**	**Elamipretide (MTP**-**131)** vs. placebo	**Mitochondrial function, apoptosis** (preserves the integrity of cardiolipin, enhances mitochondrial energetics, and improves myocyte survival during reperfusion)	Randomized, double-blind, placebo-controlled	Before PPCI, at least 15 min, but no more than 1 h, intravenous infusion at 0.05 mg/kg/hr for 1 h	300 anterior STEMI patients	IS as measured by the AUC of serum CK-MB at 24 and 72 h post-PPCI	-	Hortmann M, 2019, Eur Heart J Acute Cardiovasc Care [184]; Gibson GM, 2016, Eur Heart J. [185]
**Efficacy Study of Glucagonlike Peptide-1 to Treat Reperfusion Injury/NCT02001363**	**Liraglutide (GLP-1)** vs. placebo	**Cardiac and vascular myocytes’ function** (increased cGMP release, vasodilatation, and coronary flow through GLP-1R)	Randomized, double-blind, placebo-controlled	Before PPCI, within 30 min, subcutaneous bolus (1.8 mg) followed by maintained dose for 7 d after the PPCI (0.6 mg for 2 d, 1.2 mg for 2 d, followed by 1.8 mg for 3 d).	96 STEMI patients undergoing PPCI	The salvage index measured by CMR after 3 m post-PPCI and final IS. MACE incidence at 6 m follow-up	The final IS and serum hs-CPR were lower in the liraglutide group. During a 6-m follow-up period, no difference in MACE incidence	Chen WR, 2016, Circ. Cardiovasc Imaging [186]
**-**	**TY-51924** vs. placebo	**Inhibition of activation of the Na(+)/H(+) exchanger (NHE)**	Randomized, open label, placebo-controlled	During PPCI, intravenous injection of 10, 20, or 30 mg/kg	105 patients with first anterior STEMI undergoing PPCI	MSI as determined by SPECT at 3–5 d after PPCI	No significant results	Kimura K, 2016, J. Cardiol. [187]
**CIRCUS** (Cyclosporine and Prognosis in Acute Myocardial Infarction Patients)/**NCT01502774**	**Cyclosporine A** vs. placebo	**Mitochondrial function, apoptosis** (through an inhibitor of MPTP, actually not a blocker, but an increaser of the threshold of opening)	Randomized, double-blind, placebo-controlled	Before recanalization, an intravenous bolus injection of 2.5 mg/Kg	970 anterior STEMI patients undergoing PPCI within 12 h after symptom onset	Combined incidence of total mortality; hospitalization for heart failure; LV remodeling at 1 y post-PPCI	No significant results at 1 y- follow-up	Cung T, 2015, NEJM [148]
**DANAMI-3** (The Third DANish Study of Optimal Acute Treatment of Patients with ST-segment Elevation Myocardial Infarction)/**NCT01435408**	Ischemic postconditioning of the heart during PPCI		Randomized, double-blind, placebo-controlled	After PCI and before stent implantation 4 repeated 30 s balloon occlusions followed by 30 s of reperfusion	1234 STEMI patients with onset of symptoms within 12 h and TIMI 0–1	IS salvage index at 3 m and all-cause mortality, heart failure (postconditioning) at 2 y	No significant results	Engstrøm T, 2017, JAMA Cardiology [138]
**PROMISE** (Myocardial Protection With Adenosine During Primary Percutaneous Coronary Intervention in Pts With STEMI)/**NCT00781404**	**Adenosine** vs. placebo	**Vascular function**	Randomized, double-blind, placebo-controlled	Before PPCI, single intravenous infusion bolus of 0.45 mg/mL	201 STEMI patients undergoing PPCI within 6 h of symptom onset	IS measured by CMR at 5–10 d after AMI	Intracoronary administration of Adenoisne prior to PPCI limits IS, and has a good effect on MSI and LVEF	Garcia-Dorado D, 2014, Int J Cardiol. [188]
**PROTECTION AMI/NCT00785954**	**Delcasertib**	**Selective inhibitor of delta-protein kinase C (delta-PKC)**	Randomized, double-blind, placebo-controlled	Before PPCI, intravenous infusion of 50, 150, or 450 mg/h, continued for ∼2.5 h.	1176 STEMI patients undergoing PPCI within 6 h of symptom onset	IS as assessed by CK-MB AUC	No reduction in biomarkers of MI	Lincoff AM, 2014, Eur Heart J. [189]
**GIPS-III** (Metabolic Modulation With Metformin to Reduce Heart Failure After Acute Myocardial Infarction: Glycometabolic Intervention as Adjunct to Primary Coronary Intervention in ST Elevation Myocardial Infarction)/**NCT01217307**	**Metformin** vs. placebo	**Oxidative stress, apoptosis, mitochondrial function** (activation of the AMPK and RISK pathways, attenuation of mitochondrial dysfunction, decrease in myocardial oxidative stress, reduction in apoptosis)	Randomized, double-blind, placebo-controlled	After PPCI, oral treatment with 500 mg b.i.d. over 4 m	380 (191 vs. 189) STEMI patients undergoing PPCI within 12 h of symptom onset	LVEF measured by CMR at 4 m after STEMI	No improvement in LVEF at 4 m, nor beneficial long-term effects at follow-up of 2 y	Lexis CP, 2014, JAMA [190]; Hartman MHT, 2017, Clin Res Cardiol. [158]

Abbreviations: ACS: acute coronary syndrome; AUC: area under the curve; b.i.d.: bis in die (twice daily); CK-MB: creatine kinase isoenzyme type muscle-brain; CMR: cardiac magnetic resonance; CRP: C reactive protein; d: day(s); ECG: electrocardiogram; h: hour(s); IS: infarct size; LGE: late gadolinium enhancement; LV (EF): left ventricular (ejection fraction); m: month(s); MACE: major adverse cardiovascular events; MI: myocardial infarction; min: minutes; MMP: matrix metalloproteinase; MSI: myocardial salvage index; PPCI: primary percutaneous coronary intervention; RIC: remote ischemic conditioning; s: second (s); SOFA: sequential organ failure assessment; SPECT: single photon emission computed tomography; (N-)STEMI: (non-) ST-segment elevation myocardial infarction; t.i.d.: tris in die (three times daily); TIMI: thrombolysis in myocardial infarction; Tn: troponin; w: week(s); y: year(s).

## Data Availability

No new data were created or analyzed in this study.
